# The Complexity of Soluble Checkpoint Molecules in Modulating Immune-Mediated Diseases and Autoimmunity

**DOI:** 10.3390/ijms27167319

**Published:** 2026-08-16

**Authors:** Elias Toubi, Raeda Mubariki, Zahava Vadasz

**Affiliations:** 1 Rappaport Faculty of Medicine, Technion, Haifa 31096, Israel; elias.toubi@gmail.com (E.T.); primrosethg@gmail.com (R.M.); 2 The Proteomic Unit, Bnai-Zion Medical Center, Haifa 31048, Israel

**Keywords:** soluble checkpoint molecules, modulation, autoimmunity

## Abstract

For many decades, the regulation of immune responses, namely, the maintenance of self-tolerance and the prevention of pro-inflammatory processes, was attributed to the suppressive function of T and/or B regulatory cells. However, the field of immune regulation was recently expanded to include not only many membrane-bound regulatory molecules on immune cells but also the release of many soluble molecules playing a role in the modulation of immune responses. Soluble checkpoint molecules are the subject of many recent studies, in which they were reported to function as both regulatory and stimulatory molecules. They are involved in the pathogenesis of immune-mediated disorders, infections, and cancer, and are involved in balancing immune effector responses with regulatory reactions. Many of them have become relevant targets in treating these diseases. In this review, we chose to focus on those soluble molecules whose role is attributed to immune-mediated diseases, namely in modulating autoimmunity by enhancing mechanisms of self-tolerance and immunosuppressive signals. The understanding of these mechanisms might facilitate the development of future therapeutic approaches.

## 1. Introduction

The inhibitory function of T regulatory (Tregs) and B regulatory (Bregs) cells is fundamental for maintaining self-tolerance and the prevention of immune-mediated diseases and autoimmunity. For many decades, these cells were defined as specific subtypes of T and B cells and characterized by the expression of related markers [1,2,3]. In addition to the cell-to-cell suppressive mechanisms of effector cells, regulatory cells are producers of inhibitory cytokines such as IL-10, TGF-β, IL-2, and IL-35 [4,5,6]. In recent years, regulatory cells have been additionally defined by the expression of many immune membrane-bound checkpoint molecules. A growing body of data has recently focused on the importance of the soluble forms of checkpoint molecules. The aim of this review is to illuminate and expand knowledge on the role of soluble checkpoint molecules, specifically in immune-mediated diseases. Dysregulation of immune checkpoint pathways contributes to the pathogenesis of numerous immune-mediated diseases, including inflammatory bowel disease, rheumatoid arthritis, psoriasis, and systemic lupus erythematosus, by disrupting mechanisms of peripheral tolerance and promoting chronic inflammation. Key checkpoint molecules, including PD-1, CTLA-4, LAG-3, TIM-3, TIGIT, and TNFAIP3-regulated signaling pathways, influence immune cell activation, differentiation, cytokine production, and tissue repair. Consequently, immune checkpoint molecules have emerged as both biomarkers of disease activity and promising therapeutic targets, with modulation of these pathways offering the potential to restore immune balance while minimizing systemic immunosuppression.

### Membrane-Bound Checkpoint Molecules

The last decade has witnessed dramatic advances in the role of membrane-bound checkpoint molecules, expressed on almost all myeloid-derived cells, capable of modulating both innate and adaptive immune responses. They are a big family of molecules, some of which are inhibitory, such as LAG-3, TIM-3, and CD200, whereas others, such as ICOS and OX40, are defined as co-stimulatory. All membrane-bound molecules are widely reported to play a simultaneous role in the pathogenesis of many diseases such as infections, malignancy, and immune-mediated diseases [7,8,9]. In this review, we chose to focus on molecules primarily involved in modulating immune-mediated diseases.

**LAG-3** is a CD4+-related MHC class II binding molecule, expressed on activated and exhausted CD4+ and CD8+ T cells as well as on Tregs. The binding of LAG-3 to its ligand, MHC class II, induces T effector cell suppression via its cytoplasmic domain, thus regulating their activation, proliferation, and pro-inflammatory cytokines and finally preventing autoimmune responses. In this respect, stable MHCII-LAG-3 interaction was suggested to act as a potential therapeutic target in human diseases [10,11]. CD4+CD25-LAG-3+ Treg cells express high levels of IL-10 in response to TCR stimulation and are reported to have immunosuppressive activity both in vitro and in vivo. In a later study, CD4+CD25-LAG-3+ T cells were identified in human tonsil as a distinct Treg subset, producing high amounts of IL-10 and expressing low levels of FOXP3. CD4+CD25-LAG-3+ Tregs inhibited antibody production and demonstrated an efficient ability to induce B cell apoptosis [12,13]. The synergistic expression of LAG-3 with PD-1 is more effective in preventing autoimmunity in mice. In this respect, LAG-3-/- together with PD-1-/- in BALB/c mice induced lethal myocarditis, strengthening the concept of the importance of their co-expression in preventing inflammation [14]. The downregulation of LAG-3 expression induces persistent autoimmunity and rapid immune-mediated tissue inflammation. Alternatively, during persistent antigen stimulation, such as in chronic viral infection, LAG-3 expression is increased, leading to T cell exhaustion and decreased function [15,16]. Similar to LAG-3, **CTLA-4** is also widely reported to play a role in preventing immune-mediated responses. Naturally occurring CD4+FOXP3+ regulatory T cells express the inhibitory CTLA-4 molecule, which is essential for regulating immune-mediated responses [17,18]. The loss of CTLA-4 expression on Tregs was reported in many studies to induce systemic lymphoproliferation, T-cell-mediated autoimmunity, and the increased production of IgE. In addition, CTLA-4 deficiency affects the ability of Tregs to downregulate CD80 and CD86 expression on dendritic cells, and to suppress their antigen-presenting potency as well as the activation of T cells. Manipulating CTLA-4 expression and enhancing its inhibitory signals have been suggested as a promising strategy for the immunotherapy of autoimmune diseases [19,20]. The expression of CTLA-4 on Tregs and conventional T cells is essential for maintaining self-tolerance and T-cell homeostasis. Mice lacking CTLA-4 on both Tregs and conventional T cells were shown to lose the early onset of T-cell tolerance and develop T-cell activation and the extensive accumulation of pathogenic T cells in vital tissues [21,22]. **CD72** is a B-cell co-receptor that is expressed in all stages of B-cell development but is not expressed on plasma cells. It is a regulatory molecule that controls B-cell differentiation, particularly from naïve B cells into plasma cells, thus preventing the production of autoantibodies. CD72 is a co-receptor of the B cell receptor (BCR), which carries an immune receptor tyrosine-based inhibition motif (ITIM). Following their phosphorylation, these motifs recruit the SH2 domain and negatively regulate BCR signaling. BCR-mediated signals were shown to be increased in CD72-deficient cells, leading to autoimmunity in mouse models [23,24]. In one of our previous studies, CD72 expression on activated B cells of patients with systemic lupus erythematosus (SLE) was significantly lower than that of the normal controls. This decreased expression correlated with SLE disease activity, namely, with lupus nephritis and the higher titers of anti-dsDNA antibodies [25]. The regulatory function of CD72 in downregulating auto-reactive B cells was demonstrated by its ability to recognize Sm/RNP (a lupus self-antigen) at the extracellular C-type lectin domain, thereby preventing the production of anti-Sm/RNP antibodies. The colligation of BCR and CD72 is achieved only when Sm/RNP (a ligand of CD72) interacts with anti-Sm/RNP-BCR. The requirement of colligation with BCR may restrict CD72 to specifically inhibit the activation of anti-Sm/RNP B cells [26]. The immune checkpoints **PD-1 and PD-L1** are expressed on most immune cells, namely monocytes, DCs, B cells, and T cells. The binding of PD-1 to its ligands PD-L1 and PD-L2 induces inhibitory signals that lead to downregulation of immune-cell activation and survival, as well as the secretion of pro-inflammatory cytokines. The inhibitory function of both PD-1 and PD-L1 is highly related to their expression on Tregs and Bregs, thus maintaining the suppressive function of these regulatory cells and preventing immune-mediated diseases [27,28]. PD-1+ and PD-L1+ Bregs are identified as IL-10+ B cells, inhibited by tCD4+ and CD8+ T cell activity, and subsequently were shown to increase the function of Tregs, namely, their ability to produce IL-10 [29,30]. PD-L1-expressing B cells were assessed in the peripheral blood of rheumatoid arthritis patients. The frequency of PD-L1+ B cells was significantly lower in active RA patients than in healthy individuals. Following treatment and clinical improvement, the number of these regulatory cells was restored, and they exhibited T-cell suppressive capacity. PD-L1+ B cells were also demonstrated to be IgA+ B cells in animal models. In this regard, IgA+ PD-L1+ B cells were reported to inhibit TNF production by T effector cells and block the development of experimental autoimmune encephalomyelitis (EAE) in mice [31,32]. Decreased numbers of PD-L1+ and PD-L2+ Bregs are associated with autoimmunity; thus, the adaptive transfer of PD-L1+ B cells to EAE mice inhibited disease activity and decreased Th1/Th17 cells. CD5highPD-1+ B cells were also found to produce IL-10 following their binding to PD-L1+ B cells [33]. When these B cells were transferred to mice, they effectively inhibited CD+ T cell responses. Thus, the enhancement of PD-1 expression in mouse models was demonstrated to be beneficial in regulating peripheral T-cell responses in human and mouse arthritis [34]. **CD1d** is a regulatory molecule, highly expressed on murine IL-10+ Bregs, thus being essential for IL-10-dependent suppression of colitis. CD1d was also shown to be highly expressed on human IL-10+ Bregs and on mature CD5+ B cells [35,36]. In addition, CD1d+ Bregs were reported to secrete the regulatory cytokine TGF-β in humans and to suppress Th1 and Th17 cells, thereby playing a role in maintaining self-tolerance and preventing autoimmunity in mouse models [37,38]. In another study, CD1d surface expression was shown to be decreased on SLE transitional B cells, suggesting a role in the pathogenesis of SLE [39,40]. With all the above in mind, all membrane-bound checkpoint molecules are perfect guardians in preventing immune-mediated inflammation and autoimmunity. Their regulatory/suppressive mechanisms are well established; therefore, they are targets of already established therapeutic strategies.

Soluble forms of checkpoint receptors or ligands are produced following their cleavage from the cell membrane of immune cells. This cleavage is mediated by metalloproteinases during cell activation. The regulatory mechanisms of soluble molecules are still controversial; some of them, such as CTLA-4, remain inhibitory in their soluble form, and others, such as soluble CD72, are stimulatory. As a result, we aimed to shed light on and expand the scope of those soluble checkpoint molecules, which are emphatically involved in immune-mediated diseases.

## 2. Soluble Checkpoint Molecules

### 2.1. Soluble PD-1, sPD-L1, and sPD-L2

**Figure 1:**
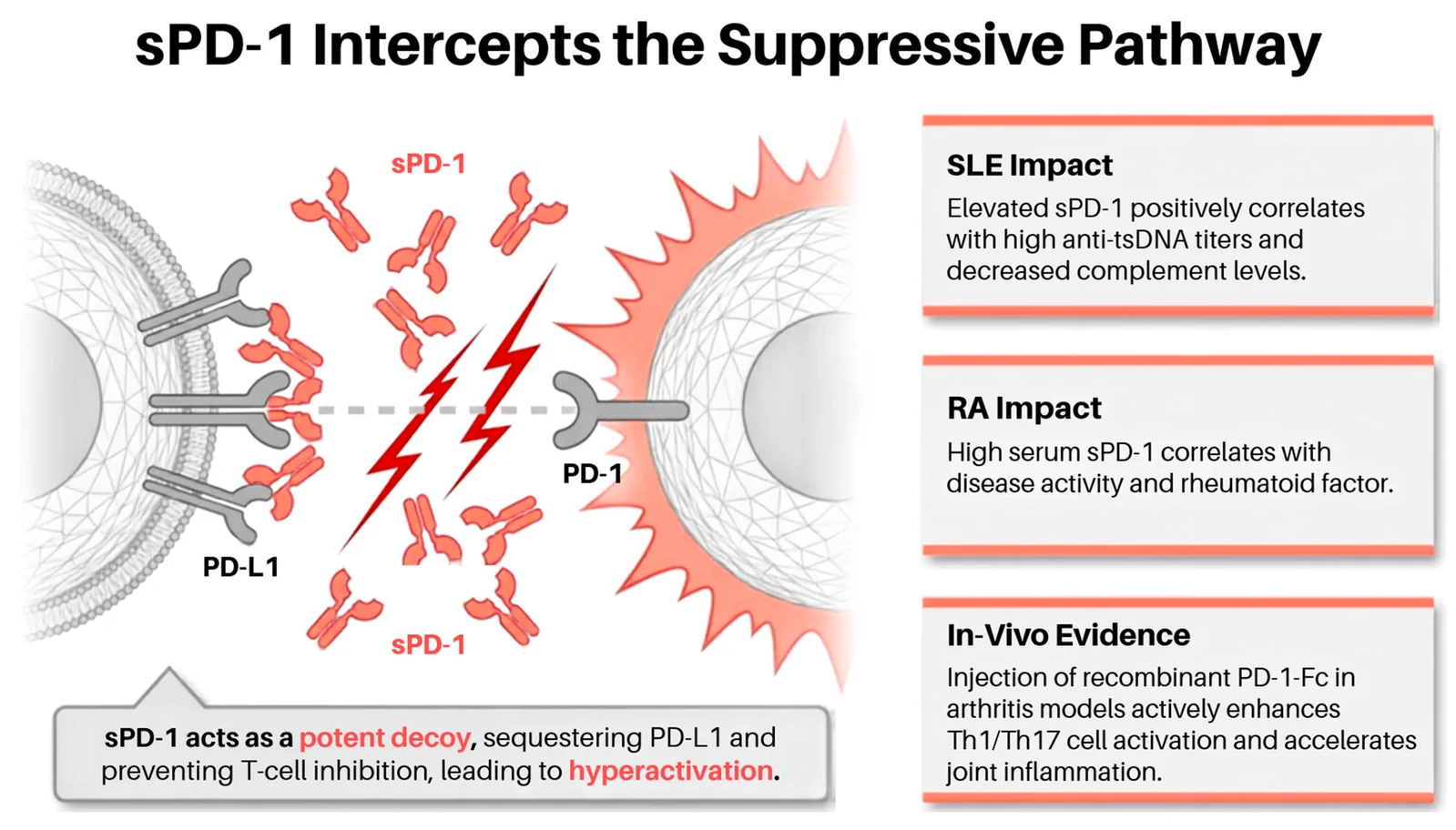
Soluble PD-1 and PD-L1,2 play a role in the pathogenesis of SLE and RA. In malignancy, blocking the PD-1/PD-L1 pathway with sPD-1 has been found to reverse T lymphocyte depletion and restore anti-tumor immunity. However, exoPD-L1 was shown to contribute to immunosuppression as a biomarker for tumor progression.

The origin of these molecules is thought to be primarily from two mechanisms: alternative mRNA splicing and the proteolytic shedding of their membrane-bound forms, expressed on activated immune cells by proteases. The role of sPD-1 and sPD-L2 is considered to increase the diversity and complexity of the inhibitory PD-1/PD-Ls pathway. However, their role in regulating immune-mediated responses is still not clear enough. Serum levels levels of sPD-1, sPDL1 and sPDL2 were recently assessed in patients with SLE. Both sPD-1 and sPD-L2 were found to be significantly higher in patients with active SLE than in healthy controls. In contrast, sPD-L1 was not increased. Increased sPD-1 and sPD-L2 were found to be positively correlated with anti-dsDNA titers and complement serum levels. Serum levels of sPD-1 and sPD-L2 were restored following clinical improvement [41]. In another study, sPD-1 and sPD-L1 levels were significantly increased in active SLE compared to healthy controls. Here, increased levels were also associated with high SLEDAI and decreased complement levels. These two studies suggested that increased soluble PDs might act as blockers/regulators of membrane PD-1 or PD-Ls, thus inhibiting their regulatory function [42]. In a later study, membrane-bound PD-L2 was found to be highly expressed on monocytes of SLE patients, whereas sPD-L2 was significantly lower in these patients than in normal controls. Lower sPD-L2 serum levels were found to correlate with lupus nephritis and joint pains. In this case, lower soluble levels may reflect their lower cleavage from membrane surfaces and, in addition, their lower peripheral inhibitory signals and poor ability to prevent immune-mediated inflammation [43]. The status of sPD-1 and sPD-L1was assessed in patients with immune thrombocytopenia (ITP). In one study, serum sPD-1 and serum sPD-L1 were found to be significantly decreased when compared with those in healthy controls. This was found to be positively correlated with platelet counts. These findings were thought to be responsible for the altered inhibitory regulation of these PDs, leading to increased T cell activation and inflammatory responses in ITP [44]. However, in a later study, controversial findings were reported in patients with ITP. Here, both PD-1+CD4+ T cells and PD-1+DCs were significantly higher in peripheral blood from ITP patients before treatment. In addition, sPD-1 was higher in ITP than in healthy subjects, correlated with IFN concentration, and negatively correlated with platelet count. In this case, the authors speculated that increased sPD-1 in peripheral blood blocked the PD-1/PD-L1 signaling pathway, allowing cell function to remain active and pro-inflammatory [45]. In line with the above findings, increased serum sPD-1 concentration was found in patients with active rheumatoid arthritis in correlation with disease activity and increased titers of rheumatoid factor. In this study, sPD-1 was proven to functionally block the inhibitory effect of membrane-bound PD-1 on T cells. An additional important finding of this study was that when recombinant PD-1-Fc was injected into a model of collagen-induced arthritis, it enhanced the activation of Th1 cells and Th17 cells as well as joint inflammation in these mice [46]. In a recent study, the role of soluble PDs in the peripheral blood of pregnant women was assessed. Soluble PD-L1 was found to be higher in pregnant women than in non-pregnant women, but serum sPD-1 levels were lower. Both findings are suggestive of a role in suppressing maternal immunity [47]. Additionally, sPD-1 and sPD-L1, as well as exosomal PD-L1, were also reported to be increased in the serum of cancer patients. In this case, blocking the PD-1/PD-L1 pathway by sPD-1 has been shown to reverse T lymphocyte depletion and restore anti-tumor immunity. However, exosomal PD-L1 was shown to play a role in immunosuppression as a biomarker for tumor progression [48]. See Figure 1.

### 2.2. Soluble CTLA-4

**Figure 2:**
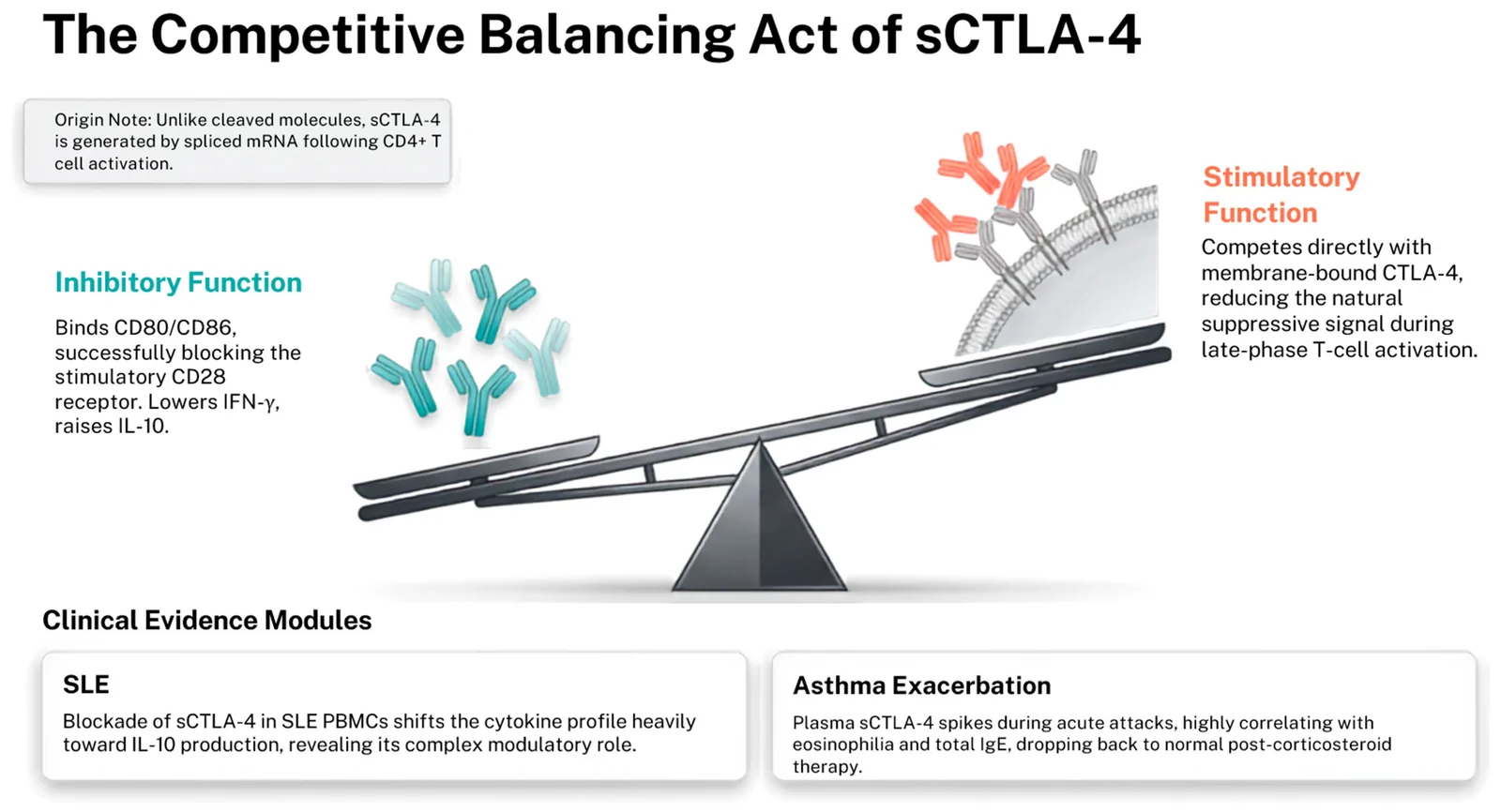
The generation of sCTLA-4 inhibits T-cell activation by competing with membrane CTLA-4. It also downregulates CD8+ cytotoxic T cell function. Increased sCTLA-4 is associated with increased inflammatory processes such as those in bronchial asthma.

Soluble CTLA-4 in peripheral blood is generated by spliced mRNA following the activation of CD4+ T cells and is usually detected at low serum levels in healthy individuals. Regulatory T cells are the most active sCTLA-4 producers in basal and inflammatory states. Increased sCTLA-4 serum levels are reported in many autoimmune diseases, including SLE, Graves’ disease, and myasthenia gravis. The biological function of increased sCTLA-4 is controversial. On one hand, it is considered inhibitory by binding CD80/CD86, blocking the co-stimulatory receptor CD28. In this case, it inhibits the secretion of IFN-γ and IL-2 and increases the secretion of TGF-β and IL-10. Alternatively, sCTLA-4 may bind the membrane form of CTLA-4, thus inhibiting its binding to CD80/CD86, reducing its inhibitory signaling. The above two scenarios are potential players that may lead to different outcomes in different autoimmune diseases [49,50]. Soluble CTLA-4 has been reported to play a role in immune regulation by enhancing Treg function. In this case, the loss of sCTLA-4 resulted in the impairment of Treg function and the ability to downregulate dendritic cell co-stimulation. Soluble CTLA-4 knockdown Tregs and diminished sCTLA-4 expression were associated with increased onset of autoimmune diabetes in transgenic mice. In an earlier study, sCTLA-4 was able to inhibit the proliferation of memory autoreactive T cell clones in a dose-dependent manner. In contrast, recombinant fusion protein CTLA-4Ig, but not sCTLA-4, suppressed naïve alloreactive T cell proliferation, indicating that sCTLA-4 and CTLA-4Ig play distinct roles in suppressing memory and naïve T cell immune responses [51,52]. Soluble CTLA-4, sCD80, and sCD28 were assessed in the plasma of SLE patients and were found to be increased in correlation with SLE disease activity. The study suggested sCTLA-4 concentration serves as a marker of SLE disease activity, without providing any mechanistic explanation for this increase. When lupus autoantigenic peptides were incubated with PBMCs of SLE and healthy controls, PBMCs from healthy donors responded to all lupus peptides by secreting IFN-ϫ and IL-17. However, PBMCs from SLE patients produced mainly IL-10. Whereas serum sCTLA-4 was similar in healthy controls and SLE patients, blockade of sCTLA-4 enhanced the cytokine profile in PBMC cultures from both groups [49,53]. Soluble CTLA-4 was assessed in the plasma of adult patients suffering from bronchial asthma and compared with that in healthy individuals. Plasma sCTLA-4 concentration was significantly higher in all asthmatic patients than in normal controls. In addition, increased levels of serum total IgE were positively correlated with increased sCTLA-4, suggesting this has a role in enhanced immune responses and the immune pathogenesis of asthma. In another study by the same group. Plasma sCTLA-4, sCD28, sCD80 and sCD86 concentrations were found to be significantly higher in asthmatic children at the time of acute attacks. Interestingly, the increased sCTLA-4 level was highly correlated with eosinophilia in these patients. Finally, sCTLA-4 and all other assessed soluble molecules decreased and returned to normal levels following corticosteroid therapy. The above findings suggest that sCTLA-4 contributes to asthma severity and acts as a marker for asthma exacerbation [54,55]. Two decades ago, the functional role of sCTLA-4 was assessed in patients with autoimmune thyroid disease. In this study, the authors demonstrated that its binding inhibited sCTLA-4-mediated immune reactivity to B7-1, suggesting that sCTLA-4 is a functional receptor. In a later study, sCTLA-4 serum levels were found to be elevated in various autoimmune thyroid diseases when compared to healthy individuals. However, this was not related to any clinical manifestation such as hypo- or hyperthyroidism, nor to serum levels of thyroid hormones. Importantly, sCTLA-4 was shown to bind its ligands CD80/CD86, thereby enhancing inhibition of early T-cell activation by blocking the interaction of CD80/CD86 with the stimulatory receptor CD28. In contrast, increased levels of sCTLA-4 were able to compete with membrane-bound CTLA-4, causing a reduction in its inhibitory signaling in the later phase of T-cell activation. These results demonstrate the controversy of sCTLA-4 signaling in the different stages of autoimmune responses [56,57]. Finally, the role of sCTLA-4 in anti-tumor immunity was also assessed. In this respect, sCTLA-4 concentration was documented to be increased in patients with breast cancer. It was speculated that increased sCTLA-4 might increase the membrane-bound-induced suppression of the anti-tumor immune response and enhance tumor growth. In a new study, the expression of sCTLA-4 by tumor cells down-regulated CD8+ cytotoxic T cell function and increased the growth of metastasis of experimental murine tumors. Blocking sCTLA-4 by isoform-specific antibodies restored the cytotoxic activity of intratumoral CD8+ T cells, resulting in a beneficial therapeutic outcome and tumor control [58,59]. See Figure 2.

### 2.3. Soluble LAG-3

**Figure 3:**
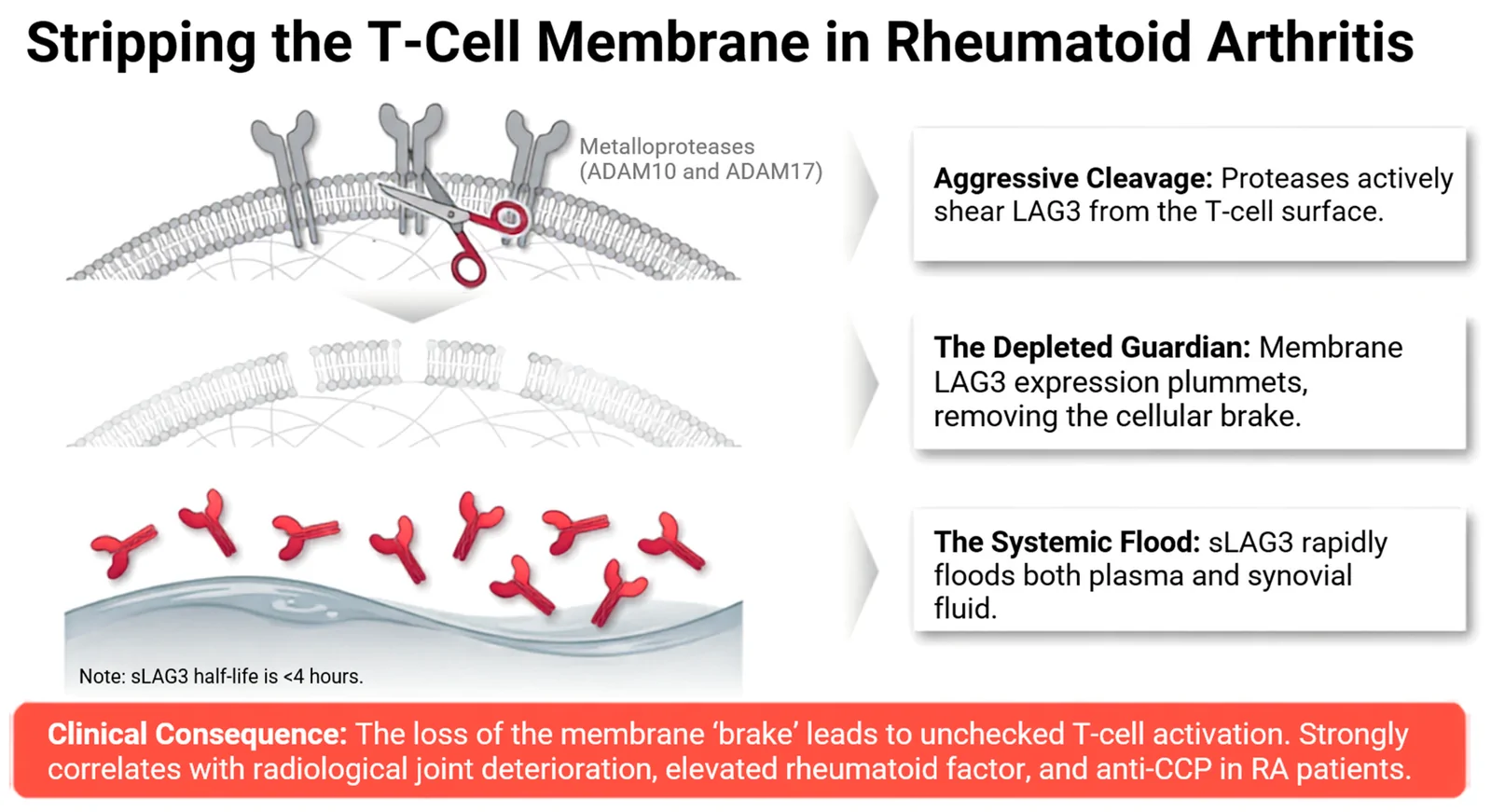
Increased sLAG3 is associated with increased disease severity of many autoimmune diseases, such as rheumatoid arthritis and myasthenia gravis. Increased sLAG3 was also associated with increased expression of antigen-specific CD8+ T cells and a better outcome of breast cancer.

**Figure 4:**
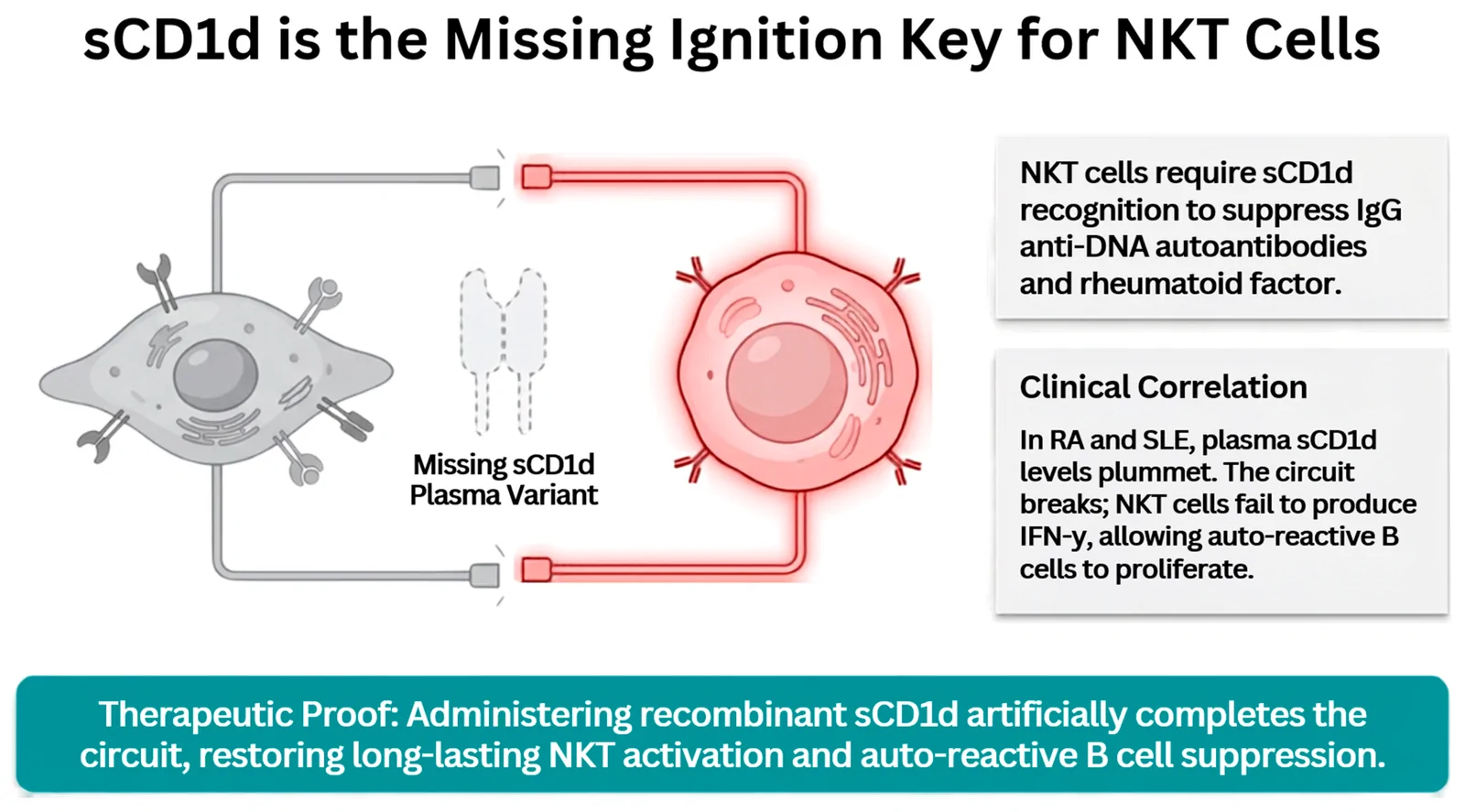
Soluble CD1d is important in controlling autoimmune diseases. Thus, low levels of sCD1d are associated with disease severity of rheumatoid arthritis and joint inflammation. In addition, sCD1d plays a role in enhancing anti-tumor immune responses.

**Figure 5:**
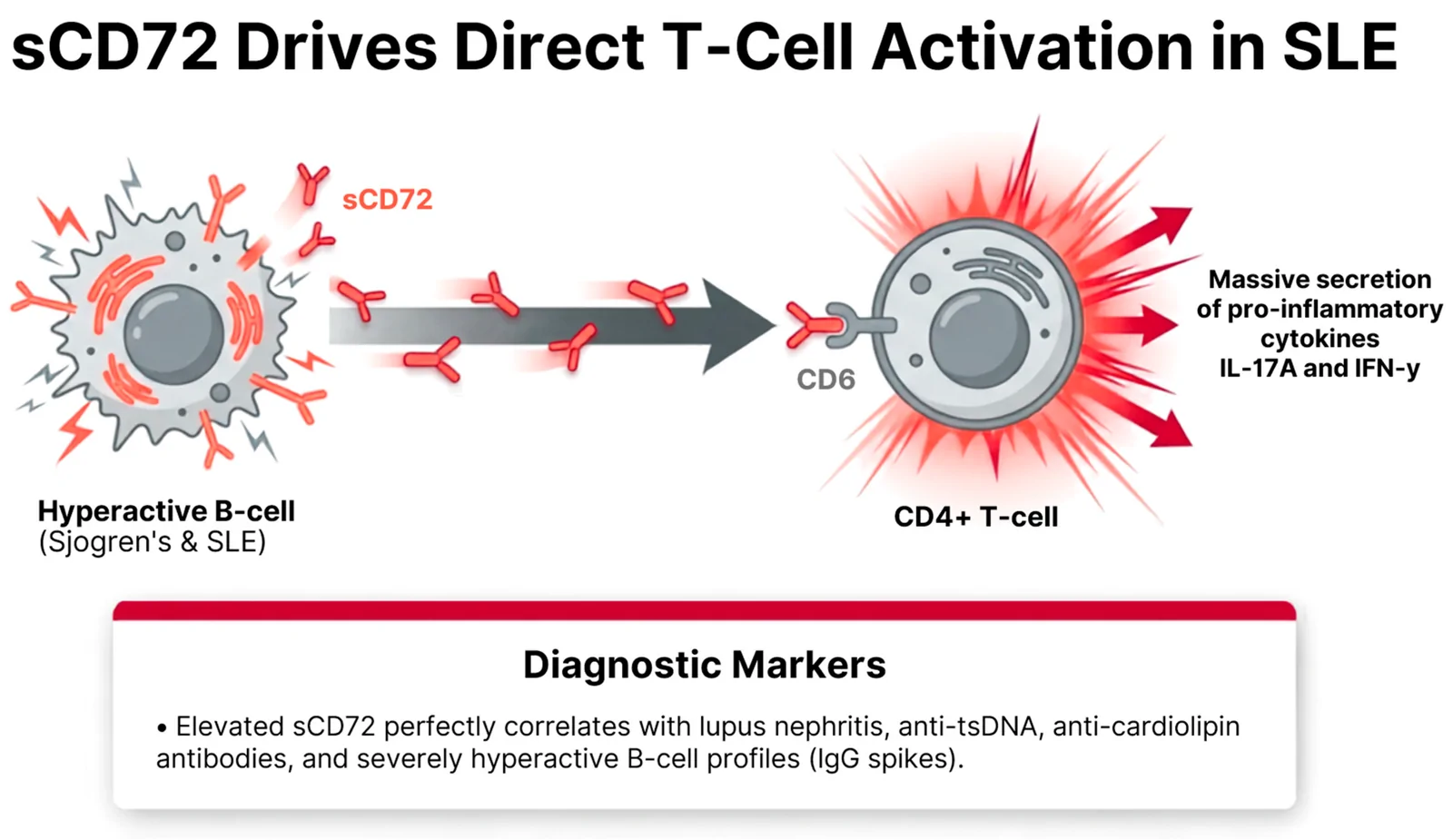
In contrast to membrane-bound CD72, soluble CD72 increases pro-inflammatory responses in both SLE and Sjogren’s syndrome.

Soluble LAG-3 is cleaved by two metalloproteases, ADAM 10 and ADAM 17, producing a 52kDa soluble form known as sLAG-3 [60]. Soluble murine LAG-3-Ig fusion protein was demonstrated to induce MHCII-mediated signaling in DCs, enhancing their activation and the production of pro-inflammatory cytokines. With this in mind, and by activating antigen-presenting cells through MHC II signaling, sLAG-3 can be used in vaccination by increasing antigen-specific T cell responses [61]. In a recent study, sLAG-3 was found to be significantly increased in the plasma of patients with early and chronic rheumatoid arthritis. Increased levels of sLAG-3 were associated with radiological defects and their deterioration over time, as well as with increased titers of rheumatoid factor and anti-CCP antibodies. In addition, sLAG-3 expression was also increased in the synovial fluid of these patients. These results could be explained by two possibilities: increased sLAG-3 is a stimulatory soluble factor that contributes to enhanced T cell activation. Alternatively, increased sLAG-3 could act as a compensatory mechanism aiming to suppress the overactivity of T and B cells, but without any success [62]. Soluble LAG-3 was speculated to bind membrane-bound LAG-3 and block the interaction of membrane LAG-3 with MHC II, inhibiting its suppressive effect. In addition, it was demonstrated that the half-life of purified sLAG-3 in peripheral blood is less than four hours and therefore does not bind to MHC II on B cells. This suggests that as sLAG-3 is increasingly cleaved from the cell membrane, it is degraded, leaving T cells with insufficient membrane LAG-3 expression, thus resulting in a decreased suppressive ability. In this case, it is still controversial whether sLAG-3 contributes to the inhibition or stimulation of immune responses [63]. Though mainly expressed on activated T cells, LAG3 expression was also demonstrated to be present on activated B cells, considered to be produced endogenously. When activated T cells were co-cultured with activated B cells, sLAG-3 was passively absorbed by activated B cells, leading to increased expression, suggesting this was T-cell dependent [64]. The status of sLAG-3 was shown to play different roles in different malignancies. High serum sLAG-3 levels were demonstrated in patients with breast cancer and were associated with longer disease-free survival. This was attributed to the increased expression of antigen-specific CD8+ T cells [65]. Alternatively, when sLAG3 was assessed in patients with chronic lymphocytic leukemia (CLL), it was found to be significantly higher in patients in whom the disease progressed compared to those in whom CLL was stable [66]. Soluble molecules were analyzed in patients with anti-neutrophil cytoplasmic antibody (ANCA)-associated vasculitis (AAV). Soluble molecules, such as PD-L1, CD80, CD86, and sLAG-3, were elevated in AAV patients and correlated with disease activity. However, sCD27 and CTLA-4 were significantly lower. Increased soluble molecules decreased following beneficial treatment and improved outcomes [67]. In a recent study, sLAG-3 plasma levels were significantly elevated in patients with myasthenia gravis, whereas membrane-bound LAG3 (mLAG-3) expression on CD4+ T cells was decreased. The addition of IL-18 to CD4+ T cell culture induced a further decrease in mLAG-3 but increased the level of plasma sLAG-3 in the supernatant. Both mLAG-3 and sLAG-3 are potential therapeutic targets in myasthenia gravis [68]. In contrast to the above findings, sLAG-3 was significantly lower in patients with coronary artery disease (CAD) than in controls. This was negatively associated with the occurrence of CAD, body mass index, and diabetes mellitus, suggesting that reduced sLAG-3 is a risk factor for developing CAD [69]. See Figure 3. ***Soluble CD1d*:** Natural killer T (NKT) cells are recognized to play a role in the pathogenesis of autoimmunity by their ability to suppress IgG anti-DNA autoantibodies, rheumatoid factor, and the down-regulation of IL-10-producing B cells. This mechanism of NKT cell activation was shown to be mediated by recognition of CD1d on B cells, particularly autoreactive B cells. In this case, CD1d deficiency was demonstrated to be associated with higher amounts of autoantibodies in mouse models of lupus. Thus, NKT-mediated inhibition of autoimmunity is CD1d-dependent, suggesting this pathway to be a therapeutic target in treating autoimmune diseases [70]. The human CD1d gene contains 8 splice variants, of which one is a soluble form (sCD1d) lacking the Beta-2-microglobulin (β_2_-m) and transmembrane domain. The level of sCD1d in the plasma of rheumatoid arthritis patients was significantly lower compared to healthy individuals. Low levels of sCD1d in RA patients were associated with decreased NK cell activation, reflected by their altered production of IFN-ϫ, thus playing a role in the pathogenesis of RA [71]. In another study, soluble recombinant CD1d was shown to identify NKT cells and inhibit their function. Blocking CD1d with specific antibodies reversed CD1d-mediated inhibition of NKT function, providing further evidence for the important interaction between NKT cells and sCD1d in preventing autoimmune responses [72]. CD1d expression on tumors activates NKT anti-tumor response, increasing their killing ability. Thus, the down-regulation of CD1d expression on tumor cells alters their recognition by NKT cells, escaping anti-tumor responses. Taking this into consideration, a CD1d-CD19 fusion protein was reported to induce in vitro activation and cytokine production by NKT cells, as well as the promotion of DC maturation, thereby controlling the growth of CD19+CD1d- tumors. In another study, sCD1d variants were assessed by quantitative reverse transcriptase PCR and found to be increased in patients with gastric cancer, suggesting that they are involved in anti-tumor responses [73,74]. The repeated injection of recombinant sCD1d into a mouse model of lung metastasis induced long-lasting NKT cell activation in association with IFN-γ secretion as well as DC maturation, thereby increasing their anti-tumor effect [75]. See Figure 4. **Soluble CD72:** The role of CD72-expressing B cells in regulating immune responses was previously reported by demonstrating the inhibitory signals provided by CD72, maintained by self-tolerance. In this respect, CD72-deficient mice exhibited lupus-like features along with increased titers of anti-dsDNA antibodies [76]. In addition to this observation, we reported that altered expression of CD72 on B cells was associated with SLE disease activity and increased SLE-related autoantibodies [25]. To assess a possible role for sCD72, we analyzed sCD72 levels in the sera of SLE patients compared with those in healthy controls. Soluble CD72 was significantly increased in 159 SLE patients, in association with lupus nephritis, anti-dsDNA, and anti-cardiolipin antibodies [77]. Increased sCD72 in SLE patients could be explained by its increased shedding and/or cleavage from highly activated B cells. In another study, sCD72 was significantly higher in the serum of patients with primary Sjogren’s syndrome than in healthy controls. Increased sCD72 was found to correlate with increased IgG serum levels, suggesting B-cell hyperactivity [78]. In this respect, the biological effect of sCD72 on CD4+ T-cell responses was assessed. Co-precipitation experiments showed that sCD72 interacts with CD6 on CD4+ T cells, suggesting a ligand–receptor interaction. It was also demonstrated that the addition of sCD72 to CD4+ T cell culture significantly increased the expression of pro-inflammatory cytokines, especially IL-17A and IFN-γ. This suggests that sCD72 is a T cell activator, presumably via binding to CD6 on activated CD4+ T cells [79]. See Figure 5.

**In summary**: Soluble checkpoint molecules are circulating forms of immune checkpoint receptors and ligands that arise through alternative mRNA splicing, proteolytic shedding, or release within extracellular vesicles. These molecules retain the ability to interact with immune receptors and ligands, thereby modulating immune responses independently of their membrane-bound counterparts.

In immune-mediated diseases, including autoimmune disorders, chronic inflammatory conditions, and immune-mediated organ damage, altered levels of soluble checkpoint molecules such as soluble sPD-1, sPD-L1, sCTLA-4, sTIM-3, sLAG-3, and sCD72 have been associated with disease activity, severity, and treatment response. Depending on their biological context, soluble checkpoint molecules may either enhance immune activation by blocking inhibitory receptor–ligand interactions or contribute to immune suppression by preserving inhibitory signaling.

**Figure 6:**
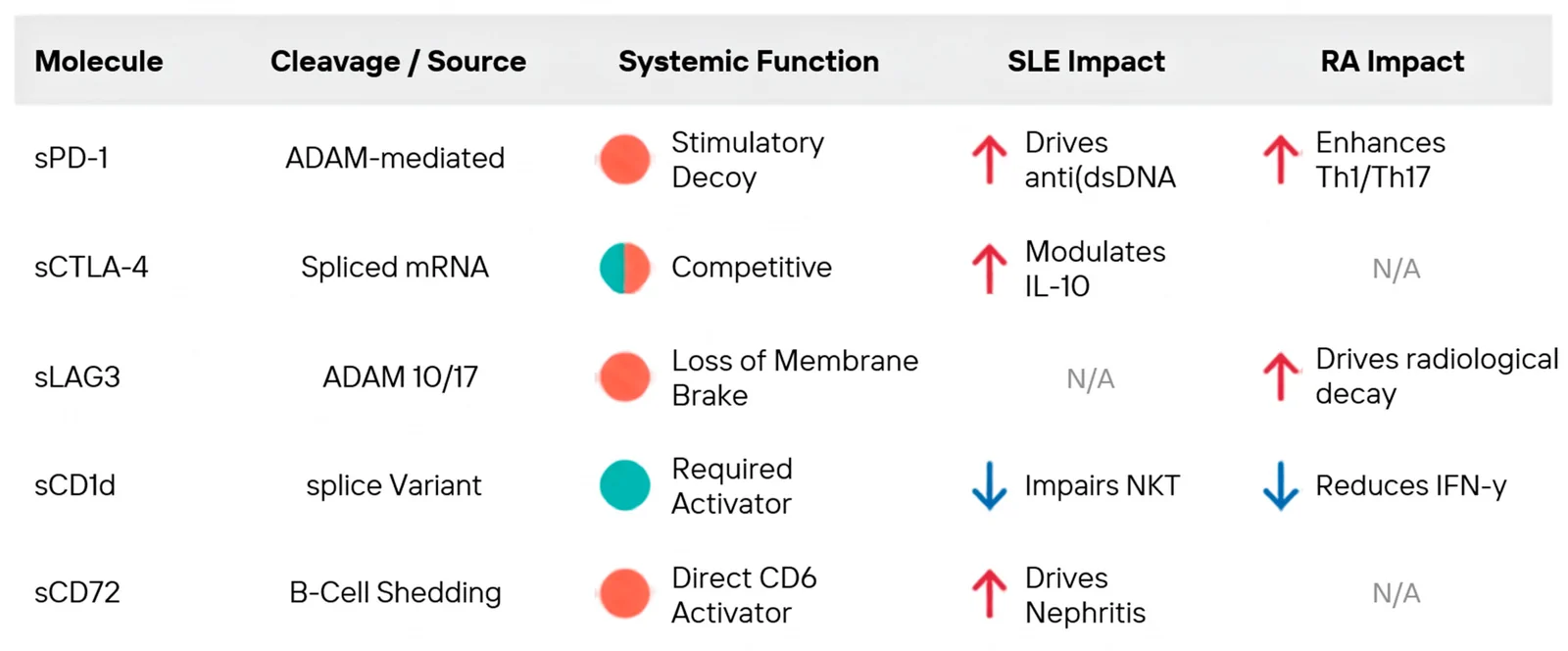
The soluble checkpoint diagnostic matrix.

Growing evidence suggests that soluble checkpoint molecules represent promising non-invasive biomarkers for disease monitoring and prognosis. Furthermore, their ability to regulate immune responses has generated interest in their potential as therapeutic targets or adjuncts to immune-modulatory therapies. However, their precise biological functions remain incompletely understood, and further studies are needed to clarify their mechanisms of action and clinical utility across different immune-mediated diseases. See Figure 6.

## Data Availability

No new data were created or analyzed in this study. Data sharing is not applicable to this article.
